# Conceptualising the separation from an abusive partner as a multifactorial, non-linear, dynamic process: A parallel with Newton’s laws of motion

**DOI:** 10.3389/fpsyg.2022.919943

**Published:** 2022-08-11

**Authors:** Daniela Di Basilio, Fanny Guglielmucci, Maria Livanou

**Affiliations:** ^1^Department of Psychology, Faculty of Health and Education, Manchester Metropolitan University, Manchester, United Kingdom; ^2^Department of Philosophy, Communication, and Performing Arts, Roma Tre University, Rome, Italy

**Keywords:** domestic violence, separation, leaving an abusive partner, Stages of Change, turning points, professionals supporting victims, post-traumatic growth

## Abstract

The present study focused on the dynamics and factors underpinning domestic abuse (DA) survivors’ decisions to end the abusive relationship. The experiences and opinions of 12 female DA survivors and 18 support workers were examined through in-depth, one-to-one, semi-structured interviews. Hybrid thematic analysis was conducted to retrieve semantic themes and explore relationships among the themes identified and the differences in survivors’ and professionals’ narratives of the separation process. The findings highlighted that separation decisions derived from the joint action of two sets of factors, the “promoters” and the “accelerators.” Whilst the “promoters” are factors leading to the separation from the abuser over time, the “accelerators” bear a stronger and more direct connection with survivors’ decision to end the abusive relationship. Despite their differences, both these factors acted as propelling forces, leading survivors to actively pursue the separation from the perpetrator. To portray the dynamic links among these factors, we propose a conceptualisation drawn from Newton’s laws of motion. Our findings also highlighted important differences in the views of survivors and support workers, as the former conceived themselves as proactive in ending the abuse, whereas the latter described the leaving process as mainly led by authorities and services supporting survivors. This study has potential implications for research, policy and clinical practice, as it suggests that far from being a linear sequence of multiple stages, leaving an abusive relationship results from a complex interplay of factors that facilitate (“promoters”) or drastically accelerate (“accelerators”) the separation process. We argue that future research should aim at improving our current understanding of the subjective and situational factors that can act as “accelerators” or “promoters” for women’s leaving decisions. Moreover, clinicians and policymakers should invest in creating interventions that aid victims to recognise and leverage promoters and accelerators, thus increasing their readiness to end the abuse.

## Introduction

According to the latest definition of domestic abuse (DA) proposed in the Domestic Abuse Act 2021 (Home Office, 2021), DA can be considered as any abusive behaviour occurring between two people aged 16 or over and personally connected to each other^1^. This definition encompasses different types of DA (physical and sexual abuse, threats, coercion, control, psychological, emotional, and financial abuse) and defines as “abusive” behaviours that may be directed at the victim and/or or perpetrated against third parties (e.g., children) connected to this latter. This definition aims at capturing the complexity of an issue that is still considered as a “global pandemic” (Wilcox et al., 2021, p. 701) and primarily affects women and girls, as a third of women worldwide have experienced DA in their lifetime. Additionally, it has been estimated that in 2020, a woman was killed by a family member every 11 min (United Nations Office on Drugs and Crime [UNODC], 2021; World Health Organization [WHO], 2021). Ending an abusive relationship remains a complex and lengthy process that seldom follows a linear timeline, as it often entails temporary breakups and episodes of reconciliation before the final separation (Anderson and Saunders, 2003; Enander and Holmberg, 2008). Moreover, achieving the separation does not imply the end of the abuse, as DA can continue and even intensify following the decision to leave (Humphreys and Thiara, 2003; Ornstein and Rickne, 2013; Zeoli et al., 2013; Hayes, 2015), often increasing victims’^2^ risk of being seriously injured or killed by their ex-partner (Campbell et al., 2003; Garcia et al., 2007; Spencer and Stith, 2020). Over the past few decades, research on survivors’ decisions to stay or leave had two main foci. The first addressed the determinants of the separation process, i.e., the pivotal *factors* playing a role in survivors’ decision to stay, leave and return to the abuser. In this domain are situated studies (e.g., Griffing et al., 2002; Anderson and Saunders, 2003; Koepsell et al., 2006; Kim and Gray, 2008; Sichimba et al., 2020; Heron et al., 2022) that highlighted important external, internal and relationship-related factors that may influence separation decisions. The second focus of DA research concerned the *process* of leaving an abusive partner, which has primarily been conceptualised as a gradual progression through multiple stages or as the product of sudden, decisive changes (“turning points”). The following sections will present a synopsis of the key findings related to these two research traditions.

## Factors influencing the separation

### External factors

Numerous studies have shown that having limited resources for economic independence can delay the separation process (Burns, 2005; Kim and Gray, 2008), whilst a situation of economic stability can facilitate it (Rhatigan et al., 2006; Clough et al., 2014). In the post-separation stage, financial difficulties can also lead to issues in finding accommodation and stable housing solutions, which in turn might promote the return to the abuser (Griffing et al., 2002; Ponic et al., 2011; Sanders, 2014). The type and quality of the support received from formal and informal sources of help, both during and after the separation, has also been identified as a relevant factor in stay/leave decisions (Taket et al., 2014; Ekström, 2015; Oyewuwo-Gassikia, 2020; Notko et al., 2022). More specifically, informal sources of support, such as friends and family members, can promote victims’ decision to leave by offering a place to stay and emotional support. Moreover, they might play an active role in encouraging survivors to adopt measures aimed at protecting them from post-separation abuse, such as pressing charges against the perpetrator (Prosman et al., 2014). However, DA literature indicated that victims who make multiple attempts to leave might experience a gradual decrease in the support received from their loved ones, and friends and family members might also withdraw from the victim if they fear the perpetrator’s retaliation (Goodkind et al., 2003; Trotter and Allen, 2009). Similarly, survivors’ experiences of formal support received by professionals, authorities and organisations can have mixed effects on their decision to leave and maintain the separation. Research shows that victims seek different types of formal help from services and professionals, including but not limited to counsellors, support groups, helplines, family doctors, police, and social services (Barrett and Pierre, 2011; Rizo and Macy, 2011; Hegarty et al., 2013; Ford-Gilboe et al., 2015). However, the support received from these sources might not be adequate, thereby delaying the separation process or leading victims to return to their ex-partner. For example, survivors’ perceptions of police support can play a role in their decision to stay or leave (Johnson, 2007; Nnawulezi et al., 2021; Couture-Carron et al., 2022). Moreover, professionals (e.g., psychologists, gynaecologists, and general practitioners) might hold stigmatising attitudes toward DA victims (Garimella et al., 2000; Peltzer et al., 2003; Baraldi et al., 2013). This may, in turn, negatively influence survivors’ perception of services (Paranjape et al., 2007; Robinson and Spilsbury, 2008; Ragusa, 2013) and enhance feelings of helplessness, isolation and vulnerability (Macy et al., 2005). Lastly, cultural and religious norms are also widely recognised as significant factors in stay/leave decisions, with multiple studies indicating that cultural and religious norms might make it harder for victims to disclose the abuse, seek help and ultimately end the abuse (Bell and Mattis, 2000; Kyriakakis, 2014; Sabri et al., 2018; Dery et al., 2022; Li et al., 2022). Conversely, however, local stakeholders (e.g., religious representatives and community members) might also provide valuable support to victims, allowing them to disclose the abuse and offering guidance during the separation process (Pyles, 2007; Shalabi et al., 2015; Sabri et al., 2018).

### Internal (personal) factors

#### Lack of acknowledgement of the abuse and use of defence mechanisms

A set of personal factors (deep-rooted in survivors’ cognitive-affective appraisal of the abuse) has also been indicated as relevant in staying/leaving decisions. For example, some researchers posited that DA survivors remain with the perpetrator as they fail to recognise the presence of abuse (Rakovec-Felser, 2014; Herman, 2015). Conversely, ending the violent relationship often coincides with the redefinition of their relationship as abusive (Anderson and Saunders, 2003; Edwards et al., 2012). In their study involving women previously in abusive relationships, Khaw and Hardesty (2007) described their participants’ process of “realization” (p. 418), consisting of a progressive acknowledgement of the abuse experienced. In another study (Enander, 2011), women who had left their abusive partners reported that their initial view of the perpetrator as a loving partner progressively subdued in favour of gradual recognition of his abuse. The acknowledgement of the partner’s duplicity (as both caring and abusive), a dichotomy described using the term “Jekyll and Hyde” (p. 36), ultimately led to their decision to leave (Enander, 2011). This evidence suggests that victims’ acknowledgement of the abuse can be remarkably influential in staying/leaving decisions. However, as some authors (Enander and Holmberg, 2008) pointed out, an in-depth understanding of the abuse often occurs after the separation, thereby making it challenging to draw direct links between victims’ recognition of the abuse and their decision to leave their partner. Moreover, survivors might adopt defence mechanisms operating a distortion of their reality and, therefore, hindering their ability to have a clear perception of the abuse (Burke et al., 2001; Chung, 2007). In turn, the decision to stay or leave might be influenced by these defence mechanisms, among which rationalisation and denial of the abuse appear to be particularly frequent (Busch, 2004; Whiting et al., 2012). Denial often characterises the first stages of the violent relationship, in which victims seem more likely to deny the existence of abusive behaviours (Edwards et al., 2012). In using denial, women might adopt a “persona of normality” (Francis et al., 2017, p. 2207), both as a survival strategy and to keep the violence hidden from others. As far as rationalisation is concerned, victims might, for example, rationalise their partner’s controlling and coercive behaviour as a sign of love and care (Chang et al., 2006; Chung, 2007) or might believe in the “good nature” of their partner, whose violence is “unwanted” and “out of their control” (Boonzaier and de La Rey, 2003). A further expression of the attempt to rationalise the abuse may consist in the minimisation of its frequency and intensity (Logan and Walker, 2004; Whiting et al., 2012). A plethora of studies (Zink et al., 2006; Enander and Holmberg, 2008; Souto et al., 2015) offered support for the role of denial, rationalisation and minimisation in stay/leave decisions. In this regard, Brown and Muscari (2010) invited researchers and professionals supporting survivors to consider their tendency to understate the gravity of the abuse experienced. This implies the need to adopt specific measures to identify denial and minimisation of DA and accurately evaluate risk, for example, by asking victims to keep a diary of their abusive experiences (Brown and Muscari, 2010).

#### Cognitive appraisal of the abuse: Victims’ self-blame and the learned helplessness hypothesis

In some cases, DA is perceived by survivors as provoked by their characteristics and/or actions and these self-blaming attitudes might be amplified by the abuser’s tendency to blame the victim for eliciting the abuse (Reich et al., 2015; Adjei, 2018; Morrison et al., 2018). In this regard, O’Neill and Kerig’s (2000) study compared a group of DA survivors still involved in abusive relationships with survivors who had left the abuser. The results indicated that women who were still involved with the perpetrator had higher scores on self-blame measures compared to survivors who had left the violent relationship. For these reasons, interventions aimed at reducing self-blame after the separation can support victims to stay free of abuse (Evans et al., 2018). Staying/leaving decisions have also been explained through the lenses of the learned helplessness hypothesis, proposed in the seventies by Seligman and colleagues (Seligman et al., 1971; Seligman, 1972, 1975; Maier et al., 1973; Rosellini and Seligman, 1975; Seligman et al., 1975). Their model postulates that when individuals learn they have little to no control over what happens to them, they gradually reduce their efforts to produce changes in their reality (Seligman et al., 1971). In line with this model, Walker (1979, 1984) suggested that women who are exposed to long-term abuse are at risk of developing learned helplessness. This might happen, for example, if survivors develop the expectation that their partner will be abusive, regardless of their attempts to reduce conflict (Clements and Sawhney, 2000). The development of learned helplessness in DA victims might make it more challenging to end the abusive relationship (Pugh et al., 2018; Estrellado and Loh, 2019; Ali et al., 2020). In this regard, Few and Rosen (2005) outlined that victims’ repeated perception of their attempts to counteract the violence as unsuccessful led them to eventually abort them. Their participants’ narrations outlined a subdued attitude and an overall “habituation” to the violence, which hampered their ability to end the abusive relationship (Few and Rosen, 2005). Despite its importance, the learned helplessness hypothesis and its application to the understanding of survivors’ staying/leaving processes received considerable criticism. Indeed, attributing learned helplessness to survivors implies considering them as “trapped” in the abusive relationship and passively accepting the circumstances (Dunn, 2005), i.e., a situation resembling a “psychological paralysis” (Gondolf and Fisher, 1988, p. 10). On the contrary, far from being passive, victims often plan strategies to leave and make multiple attempts to end the abusive relationship (Scheffer Lindgren and Renck, 2008; Moe, 2009; Meyer, 2012) Moreover, as Peled et al. (2000) noted, women’s staying can be a deliberate choice and not necessarily a consequence of their perceived impossibility to leave.

### Relationship-related factors

#### Violence escalation, survivors’ fear, and the role of risk assessment

Violence escalation and survivors’ fear of the abuse seem to have an ambivalent role in stay/leave decisions. Whilst they can be potential catalysts for leaving (Scheffer Lindgren and Renck, 2008; Bostock et al., 2009; Gharaibeh and Oweis, 2009; Estrellado and Loh, 2019), they can also delay the separation, due to victims’ fear of the partner’s reaction to separation attempts (Kim and Gray, 2008; Cravens et al., 2015; Ivany et al., 2018). When considering the possibility of staying, leaving or returning, victims engage in risk assessment and safety planning to predict possible dangers linked to their decisions (Connor-Smith et al., 2011; Gonzalez-Mendez and Santana-Hernandez, 2014; Wood et al., 2021a). These processes are usually mediated by formal services (Stanley and Humphreys, 2014; Robinson et al., 2018; Youngson et al., 2021), but there is increasing evidence that survivors independently engage in risk assessment and safety planning, even before seeking support against DA (Martin et al., 2000; Macy et al., 2005; Connor-Smith et al., 2011; Wood et al., 2021b). This suggests that survivors’ stay/leave/return decisions are based on an evaluation of the risks they would face, although there seems to be no consensus on the accuracy of their risk assessment. Indeed, some studies suggested that survivors are usually able to predict risk levels with great precision, based on factors such as violence escalation or changes in the perpetrator’s behaviour (Heckert and Gondolf, 2004; Cattaneo et al., 2007; Bell et al., 2008; Connor-Smith et al., 2011). Other authors, however, pointed out that survivors’ judgement can be clouded by a variety of factors, such as optimistic bias (tendency to perceive negative events as unlikely to happen) or the presence of symptoms of mental illness and psychological distress (Harding and Helweg-Larsen, 2008; Helweg-Larsen et al., 2008; Vitek et al., 2018; Sinclair et al., 2020).

#### Feelings of love and attachment to the partner

Love and commitment toward the perpetrator can play a pivotal role in delaying the separation process (Truman-Schram et al., 2000; Donovan and Hester, 2010; Eckstein, 2011). Conversely, changes in romantic feelings for the perpetrator have often been connected to the decision to end the abusive relationship (Rhatigan et al., 2006; Enander and Holmberg, 2008). Nonetheless, conceptions of victims as inclined to “romanticising” their relationship (Papp et al., 2017, p. 100) fail to capture the complexity of emotional bonds in violent relationships (Fraser, 2003). Indeed, victims might be aware of the violence yet remain with the perpetrator as they feel that love and abuse are intertwined and that violence is somewhat “the harm of romantic love” (Hayes and Jeffries, 2013, p. 67). In this regard, the theory of traumatic bonding (Dutton and Painter, 1981, 1993) posits that the coexistence of the perpetrator’s caring attitude and their violence cements a dysfunctional relationship between abuser and victim, from which it can be difficult to break free.

### Children’s safety and well-being

There is a general consensus in DA research that the presence of children represents a double-edged factor, both promoting and hindering the separation process. Indeed, the attempt to safeguard children from abuse might promote women’s decision to leave (Scheffer Lindgren and Renck, 2008; Lacey et al., 2013; McDonald and Dickerson, 2013; Katerndahl et al., 2019; Heron et al., 2022). Nonetheless, fear for their children’s safety during and after the separation process might lead women to stay with, or return to, the perpetrator (Levendosky and Graham-Bermann, 2001; Haight et al., 2007; Herrero-Arias et al., 2019). Also, mothers might delay the separation process to avoid leaving their children behind, as some shelters do not accept large families, adolescents or boys (Moe, 2007). Further complexity in mothers’ decisions derives from the perceived stigma they might experience, regardless of whether they stay or leave. As Saunders and Oglesby (2016) pointed out, mothers who stay in violent relationships might be accused of not safeguarding their children, whilst mothers who leave may face other types of stigma, such as being labelled as “unfit mothers” if they seek post-separation support. Lastly, the presence of children with the perpetrator and the consequent child custody rights often expose women to DA even after the separation (Humphreys and Thiara, 2003; Beeble et al., 2007; Harrison, 2008; Hayes, 2012), rendering the victims’ healing process harder to achieve (Zeoli et al., 2013).

## The separation process: Sequential stages or turning points?

As mentioned above, some DA studies focused specifically on the *dynamics* of the separation process. In general terms, these studies could be clustered into three different groups, depending on their conceptions of the separation process. Some studies (Frasier et al., 2001; Cluss et al., 2006; Edwards et al., 2012; Reisenhofer and Taft, 2013; Zapor et al., 2015) described the leaving process as a sequence of stages, drawing from Prochaska and DiClemente’s Transtheoretical Model of Change (TTM) – often referred to as the “Stages of Change (SOC) Model” (DiClemente and Prochaska, 1982; Prochaska and DiClemente, 1982, 1983, 1986). The SOC stages encompass a continuum that goes from a stage of precontemplation (in which there is no intention to change), to action (i.e., the stage in which the desired change is implemented) and lastly, maintenance (DiClemente and Prochaska, 1982). A different definition of the separation process has been offered by studies (Chang et al., 2010; Catallo et al., 2012; Murray et al., 2015; Estrellado and Loh, 2019), conceptualising leaving decisions as a result of “turning points,” i.e., events that redirect the individual’s life path (Elder, 1985). Lastly, in the third group, there are studies (Chang et al., 2006; Khaw and Hardesty, 2007; Childress et al., 2021) that attempted to combine the SOC model with the concept of “turning points,” to develop a more nuanced understanding of the separation process. Nevertheless, to date, there is a lack of a comprehensive understanding of the separation process and more efforts are needed to merge our knowledge of the factors (the “what”) and the processes (the “how”) underpinning the separation from an abusive partner. Therefore, this study aimed to contribute to bridging this gap, exploring stay/leave decisions from both a component- and a process-oriented perspective. Furthermore, the vast majority of studies (e.g., Baly, 2010; Bowstead, 2015; Crossman et al., 2016; Khoury and Wehbi, 2016) investigated the leaving process only from survivors’ perspective, whilst valuable insights may derive from professionals supporting victims during the transition to an abuse-free life. Therefore, the current study aimed to explore the separation journey as portrayed in the accounts of both DA survivors and support workers. In particular, these professionals have been chosen as they have direct contact with survivors in the various health and social care settings they work in Bourassa et al. (2008), Heffernan et al. (2012), Lessard et al. (2014), and therefore, are likely to have first-hand knowledge of the separation dynamics.

## Materials and methods

### Participants and recruitment

The recruitment for the current study was conducted in two stages. The first stage included 12 participants aged 26–67 (*M* = 44.4), all females. All were mothers except one, and 9 out of 11 mothers had children with the abusive partner they separated from, with the remaining two having children from previous relationships. The majority of the survivors identified as White British (7; 58.3%), with two Asian (16.6%), one Black Caribbean (8.3%), and two survivors who described their ethnicity as mixed (16.6%). They were recruited *via* a United Kingdom-based DA charity providing a range of services to DA victims, including but not limited to counselling, housing advice and organisation of social groups and events for survivors. After obtaining authorisation from the charity manager, the Principal Investigator (DDB) conducted recruitment *via* several visits to the service over a period of 5 months, during which potential participants (service users) were approached and information about the study was provided. Following guidelines on recruitment of vulnerable participants (Shedlin et al., 2011; Sutherland and Fantasia, 2012; Ellard-Gray et al., 2015), this initial period of engagement with service users allowed the building of rapport, for example, by creating opportunities to discuss the study in lay terms (e.g., referring to the interviews as “conversations”). To be involved in the study, participants had to be women, aged 20 years–old or older and have a history of being in an abusive relationship for at least six consecutive months, but not being in an abusive relationship at the time of the interview. This latter criterion was motivated by the nature of the study, as its primary focus was the separation process. Further information on the characteristics of the sample recruited in stage I is reported in Table 1.

**TABLE 1:** Characteristics of the stage I sample (DVA survivors).

Participant	Age at the time of the interview	Duration of the abusive bond (years)	Average time passed since the final separation from the perpetrator (years)	Other abusive relationships prior to the last one
1	40	20	4	No
2	46	7	2 weeks	Yes (one other partner)
3	42	11	1	No
4	51	19	2	No
5	26	1	4	No
6	55	4	24	Yes. Her father was abusive toward her and her mother
7	67	23	25	Yes (one other partner)
8	48	14	14	No
9	36	8	4	No
10	39	22	3	No
11	35	6	10	No
12	48	2	2	Yes (one other partner)

The second stage of the study was conducted with the participation of 18 support workers. In the United Kingdom, “support worker” is a broad term describing anyone “employed to foster independence and provide assistance in areas such as communication, employment, social participation and who may take on tasks in respect of advocacy, personal care and learning” (Manthorpe and Martineau, 2008; p. 7). In the context of DA, this definition includes professional figures such as independent domestic violence advisors, outreach and refuge support workers. The support workers involved in this study were all women aged 24–67 (*M* = 41.1). Most of them identified as White (British [13; 72.2%] and White other [1; 5.5%]), followed by Asian (3; 16.6%) and mixed/multiple ethnic groups (1; 5.5%). Overall, both samples reflected national figures on the different ethnic groups populating England and Wales, with most recent data confirming a substantially stable prevalence of people identifying as White, followed by Asian/Asian British, Black/Black British, and mixed/multiple ethnic groups (Office for National Statistics [ONS], 2019). The support workers interviewed were employed in different charities and organisations supporting DA victims located in Northwest England and West Midlands. At the time of the study, they had been in the role of support workers from a minimum of 2 years to a maximum of 10. To be included in the study, they had to be 18 years old or over and in a support worker role for more than 6 months prior to the interview. The current study included only support workers with relevant experience in helping DA victims pre– and post-separation. Support workers without such experiences (e.g., whose role was to provide brief advice through DA helplines) were not invited to participate. These criteria were included in the email sent to the managers of the organisations and charities contacted, so that only the support workers meeting the study inclusion and exclusion criteria were invited to participate in the study.

### Procedures

The data were collected in both stages of the study using face-to-face, semi-structured interviews. Before agreeing to the interview, participants in both stages read a detailed Information Sheet (which was provided in paper form in stage I and *via* email in stage II) and signed an Informed Consent Form. All participants were offered the opportunity to ask questions about the study by contacting the PI *via* email before deciding whether to take part and they were informed of their rights to withdraw from the study and to withdraw their consent to the use of their data before the stage of data analysis. The interviews were conducted in English, audio-recorded on a digital voice recorder and transcribed verbatim by the first author, who listened to each interview recording multiple times to check the accuracy of transcription. The interviews lasted from 1.5 to 2.5 h and participants received no compensation for participation. After the interviews, both survivors and support workers were provided with a paper copy of a debrief sheet containing information on how to get further psychological support, in case they experienced any psychological distress during or after the interview. Additionally, stage I interviews were conducted on the premises of the DA charity the survivors were recruited from to ensure that support was readily available if needed. According to the criterion of data saturation (Faulkner and Trotter, 2017) recruitment continued until no new themes emerged and the existing ones were saturated. More specifically, in line with the principles proposed by Francis et al. (2010), a minimum sample size for initial analysis (10 participants for each stage of the study) was set. Although there are no standardised guidelines for selecting a suitable sample in qualitative research (Baker and Edwards, 2012; Vasileiou et al., 2018), there is consensus that when deciding on sample size, qualitative researchers should refer to studies that used the same research design and wherein data saturation was achieved (Onwuegbuzie and Leech, 2007). Despite the paucity of studies on DA involving both professionals and survivors, some of them (e.g., Rose et al., 2011; Trevillion et al., 2012; Bradbury-Jones et al., 2014; Francis et al., 2017) served as relevant guidance to set a minimum sample size. Additionally, a stopping criterion was decided upon, i.e., the number of interviews to be conducted “without new shared themes or ideas emerging, before the research team can conclude that data saturation has been achieved” (Francis et al., 2010; p. 1234). Following these principles, data collection ended when the minimum sample size was obtained and saturation was achieved. The study was granted ethical approval by the Ethics Committee of the Manchester Metropolitan University (reference number IDS PGR 14/5–1) and ethical principles related to the protection of participants and their data (including preserving survivors’ anonymity and the confidentiality of their data) were followed throughout the study.

## Analysis

The transcripts were analysed using Thematic Analysis (TA) following the guidelines described by Braun and Clarke (2006, 2012). In stage I, inductive analysis was performed by the first author, who coded interview transcripts by hand, and the research team jointly discussed the developing themes; any discrepancies were assessed and negotiated until there was agreement on the final themes. In stage II, the transcripts were analysed with a hybrid approach comprising both inductive and deductive TA, in line with evidence (Fereday and Muir-Cochrane, 2006; Joffe, 2012; Xu and Zammit, 2020) indicating that hybrid TA allows to give meaning to raw data using pre-existing categories, whilst still granting a comprehensive, data-driven exploration of participants’ subjective experiences. Deductive TA was initially used by referring to a “codebook” of themes identified in the survivors’ accounts, which served as a general interpretative framework to orientate the analysis process. This allowed for the appreciation of similarities and differences in the way both groups described the separation process and stay/leave decisions. Subsequently, the support workers’ accounts were analysed further using inductive TA, to capture concepts and nuances that may not have emerged in the survivors’ narratives. As a result, the initial themes and sub-themes were modified and enriched to portray the multiple voices of participants and their views and experiences of the separation process. The hybrid approach to TA utilised in stage II was facilitated by the use of the computer-assisted qualitative data analysis software (CAQDAS) NVivo (version 11). Digital copies of the transcripts were uploaded on NVivo, and themes were applied (“nodes” on NVivo) so that each node contained all the codes semantically related to it. A reflexive approach was adopted throughout the analysis. The need to be reflexive for qualitative researchers using CAQDAS implies being aware of the potential influence that the software used could have on the ways data are handled (Woods et al., 2016). In order to be reflexive, the nodes (themes) created on NVivo were regularly checked for accuracy, consistency and representativeness. Moreover, they were iteratively revised as a result of inductive TA, which modified the initial themes applied. In both stages of analysis, all transcripts, reflexive accounts of data interpretations, field notes and developing conceptualisations of codes and themes were maintained throughout to ensure reliability and provide a clear audit trail from raw data to interpreted results (Shaw, 2010; Nowell et al., 2017). The analytic process yielded four over-arching themes, each related to different aspects of stay/leave/return decisions (“*staying: the habituation to the abusive reality*,” “*leaving: swinging before the jump*,” “*returning: the interplay of feelings and necessity,”* and “*preventing the return: rebuilding the self”*). Each overarching theme encompassed multiple sub-themes that reflected the views, opinions and experiences of survivors and professionals. The current work will solely focus on the overarching theme related to the separation process and its related subthemes, which are described below and illustrated with exemplary quotations.

## Results

### The dynamic forces underpinning the separation: “Promoters” and “accelerators”

The separation process emerged as facilitated by two types of factors: “the promoters” and the “accelerators.” The former acted over a longer timeframe (e.g., months or years), leading survivors to gradually consider the possibility of ending the abusive relationship. However, the “promoters” did not seem to have a direct connection with the decision to leave the perpetrator. This final step appeared to be more directly linked to the second order of factors, “the accelerators,” which were described by survivors and support workers as the triggering factors leading survivors to take action to end the abuse. In all the participants’ narratives, “promoters” and “accelerators” were defined either as subjective factors (e.g., victims’ feelings of fear) or as situations and events (e.g., particularly violent DA episodes).

#### “Promoters”

##### Increased awareness of the dynamics of the abuse

The survivors’ desire to end the abusive relationship was elicited and intensified by the maturation of a deeper understanding of the abuse they experienced. For example, survivor three explained that when her medication was reduced, her overall awareness increased, enabling her to recognise that her partner’s behaviour could be framed as abusive.

*S: I was more conscious, and I was more*… *aware of what was going on around me, and*… *I–I knew what was*… *right and what was wrong, and*… *that what he was doing wasn’t*… *he–he didn’t love me, it–it was just –he just wanted to control me*. S3, p. 24, ll. 525–529.

The support workers also acknowledged the importance of victims’ awareness and understanding of the abusive dynamics in promoting the separation. Many of them described their efforts to promote victims’ consciousness of the abuse, for example, by questioning their “justifications” for it.

*SW: Women will kind of say: “Well, it’s not his fault, because of this, this and this.” And then I would say: “Well, okay. So how does he ‘function’ then?”(*…*)Because if he was like that with everybody, there’d be no function, and he wouldn’t be able to work* (…). SW11, p. 2, ll. 38–45.

However, with few exceptions, the support workers highlighted that survivors seldom develop an awareness of the abusive dynamics without the help of formal services. DA survivors were described as frequently unaware of the abuse or inclined to minimise or deny it. On this aspect, survivors’ and support workers’ accounts diverged. For survivors, the acknowledgement of the abuse was a gradual process stemming from their reflections on their partner’s behaviour. For support workers, the survivors’ increased awareness was primarily an outcome of the professional support they received from different services.

##### Formal and informal sources of external support

Survivors seldom disclosed the abuse to others while it was happening, and thus, only a few of them identified comments and suggestions from family and friends as a factor that promoted their decision to leave. Similarly, only a few support workers mentioned that informal sources of support, such as friends or neighbours, can act as “promoters” of survivors’ decisions to leave. Among them, though, support worker three mentioned that neighbours could play an important role in enabling survivors to consider leaving.

*SW: She might disclose to a neighbour and then obviously the neighbour then, you know, feels that they may have to protect her and become, you know, a little bit closer* SW3, p. 3, ll. 62–64.

Different formal sources of support (e.g., police and DA organisations) were mentioned in both survivors’ and support workers’ narrations. However, the majority of the survivors interviewed reported having accessed these sources only after the separation. Thus, the formal support they received did not emerge as a strong promoter of the decision to leave. Conversely, for support workers, formal support (particularly if provided by DA organisations) was described as highly relevant in promoting leaving decisions. This is evident in the following excerpt from support worker three’s interview.

*SW: My job is to get them rehoused, my job is to find accommodation, so a lot of the women [who] have now left this- you know, have left the relationship, would still be there if I– if we hadn’t managed to get accommodation for them. So, I think it’s a massive key- key role in it*. SW3, p. 38–39, ll. 1018–1023.

##### Escalation of the abuse

The escalation of the abuse was a crucial promoter of the decision to leave the perpetrator, as survivor six highlighted:

*S: I thought– I–I’ve sort of analysed it and thought: “Each attack has got worse, first it was a slap, then it was a push up the wall, then it was ramming your head up the wall, then it was*… *a punch in the face and a black eye, then he’s finally getting on top of you and holding his hand over your mouth like he wanted me to die.*” S6, p. 22, ll. 496–502.

It is worth noticing the “slow rhythm” that characterises the factors labelled as “promoters.” In this excerpt, survivor six described a gradual crescendo of the violence, which eventually led her to end the abusive relationship. The support workers also considered the increasing intensity of the abuse or new emerging forms of DA to be factors that can slowly pave the way for leaving.

*SW: A lot of women will say to me, “Oh he’s never hit me.” You know, “He’s never hit me, but he now controls the money. Whereas before, he used to just shout and swear, now he controls my money, now it’s–,” so I think as things get worse, this– you know they start comp– a–all I mean, not everybody. But over time, um, I think they kind of look like sort of like in hindsight– think, “Well he didn’t use to do this.”* SW12, p. 1, 12–19.

##### Increase in survivors’ independence and self-confidence

Some events (e.g., a brief separation from the abuser) appeared to be beneficial for survivors’ sense of independence and self-confidence and therefore, ultimately encouraged some of them to leave. For example, during a period away from her partner, survivor two reported becoming more aware of her ability to take care of herself and this facilitated her subsequent decision to leave.

*S: I sat there and I just thought: “How can I– (pause)*… *Can I–can I be financially independent of him”? And rather than being scared of it, I embraced it; and I just thought*…(…) *I’m gonna do this, I’m gonna*… *I’m gonna –I’m going to be financially independent of him, I will*. S2, p. 82, ll. 1764–1770.

The support workers also considered increased confidence and independence to be important promoters of the separation and highlighted how they could stem from different aspects of the survivors’ lives, such as returning to work.

*SW: And they start to return to work after children have gone to school and things–* (…) *and they get a bit of independence, and that can be a factor*. SW5, pp. 1–2, ll. 21–25.

However, for most of the support workers, this increase in self-confidence was connected to the support survivors received from DA services. Therefore, rather than seeing self-confidence and increased independence as deriving from survivors’ efforts to emancipate themselves, they described these dimensions as a byproduct of the help received by DA professionals and organisations.

##### Desire to protect children from the effects of domestic abuse and the intergenerational transmission of violence

For DA survivors, another relevant promoter of the separation was the desire to protect their children from the physical and psychological consequences of being involved and/or witnessing the abuse. Interestingly, almost all survivors who were mothers reported that an important promoter for leaving was the risk of intergenerational transmission of violence (IGT), which may have affected their children if they had remained in the abusive household. For example, survivor nine explained that she wanted to protect her daughters from the possibility of internalising dysfunctional models of intimate relationships, in which DA is considered as acceptable.

*S:* (…) *because my*… *two daughters, the older ones*… (…) *were coming like in teenage years, and I just thought: “It’s–*… *They’re seeing things and hearing things that they don’t need to see and hear,” and I don’t –I didn’t want it to affect their lifestyle growing up*. S9, p. 53, ll. 1132–1137.

A similar account was offered by one of the support workers:

SW: (…) *and [they] then decide to protect the children when they witness, um, you know- older girls witnessing what dad’s like for example* (…) *Don’t want them growing up thinking this is the way that she should be treated when she is in a relationship um–you know- “This is not normal, I don’t want them thinking this is a normal situation”* (…) *Or boys mimicking what dad does*. SW4, p. 24, ll. 549–558.

Additionally, survivors and support workers mentioned that an important promoter for leaving is connected to mothers’ increased awareness of the detrimental cognitive, emotional and behavioural effects of DA exposure on their children. For example, a support worker mentioned that some mothers consider leaving when they start noticing issues in their children that are linked to their ongoing exposure to DA.

*SW:* (…) *[it’s] noticing the effect that it’s having on the children whether it’s, you know, poor performance in education or children mirroring behaviours* (…) *of a partner or regressing, things like bedwetting or things like that*. SW16, p. 2, ll. 25–30.

Another support worker remarked on the importance of this promoter by saying that, at times, the effect of DA on children represents the primary motivation that leads survivors to consider leaving the abusive relationship.

*SW*: P: *So, um, I’ve had a lot of survivors who will constantly say that they– that they know they’re in an abusive relationship, but they don’t want to do anything about it and they’re happy, and then when it’s pointed out to them, the effect it’s having on the children, I think that’s when they start– it sort of triggers, um, “It’s not just affecting me now, it’s affecting them.”* SW6, p. 1, ll. 9–15.

#### “Accelerators”

The term “accelerator” was chosen to indicate factors that were mentioned by both survivors and support workers as directly related to the separation. The accelerators are particularly intense subjective and/or situational factors that act as triggers for the leaving process as they create an insoluble rupture in the balance of the abusive relationship, thus priming the process of leaving the perpetrator.

##### Particularly violent domestic abuse episode

More than half of the survivors interviewed reported that a particularly violent DA episode acted as a trigger for leaving the abuser. For example, survivor two had left her partner and returned to him several times until a particularly intense episode of abuse occurred, which led her to leave him permanently.

*S: And then he pushed me down the stairs* (…) *and that*… *was the final straw [voice broken from crying]*. S2, pp. 77–78, ll. 1665–1667.

Similarly, in the support workers’ accounts, a severe episode of abuse could accelerate the leaving process. Support worker three reported an example from one of her client’s experiences:

SW: *But this one particular occasion, he beat her up that bad with a hammer um, she miscarried, uhmmm, so that was her, you know, ch– that was her trigger*. SW3, p. 37, ll. 967–969.

##### Perception of the abuse as unbearable

The feeling that the abuse had become intolerable led some of the survivors to end the abusive relationship. For example, survivor eight reported the sense of being exasperated by the abuse she was experiencing. The impossibility to tolerate the violence (*I couldn’t bear it any longer*, S8, p. 19, l. 414) led her to tell the abuser that she *had enough*, p. 21, ll. 461–462 of his abuse and wanted to end their relationship. To describe her inability to tolerate the abuse any further and the state of profound prostration she was experiencing, survivor six used the word “breakdown”; *I came close to a breakdown* (…) *to be honest with you*, S6, p. 26, ll. 581–582. Some support workers also reported that survivors might leave when they start perceiving the abuse as unbearable. For some of the women interviewed (survivors and support workers), the perception of the abuse as unbearable was linked to an escalation of the violence. However, in some cases, no noticeable changes in the abuse motivated survivors’ feeling that the violence had become intolerable. In these cases, this perception was described as deriving from “internal changes,” for example, a protracted state of emotional exhaustion. In this regard, a support worker reported: SW: *They feel that they’re at the bottom anyway, there’s nothing for them* (…*). The–they’re finished, they can know they’ve got nothing, they are*… *exhausted* (…) *They are wiped out, they are finished*. SW1, p. 53, ll. 1186–1192.

##### Fear for their life and safety

Survivors often reported having experienced intense abuse and life threats several times before the emergence of fear. In some way, the abuse had become an integral component of their relationship, and therefore, some of them did not feel their life could have been in danger. Nevertheless, sudden changes in the partner’s abuse (e.g., the onset of new forms of abuse) could worsen survivors’ fear for their safety and thus accelerate the separation. For example, survivor eleven narrated an episode in which her partner threatened to kill her, which immediately triggered the leaving process.

*S: He–he went to bed and then he says: “When I get up” –he says –“You’ve had it this time” –he says– “I’m deadly serious” –he says– “I’m gonna kill you.” So when he was in bed (pause)*… *–I kept checking to see–you know– up the stairs, if I could hear anything* (…) *I just grabbed my clothes, grabbed my post office book* (…) *with a bit of money in, and just ran.* S11, pp. 6–7, ll. 127–137.

The support workers’ narrations were in line with the survivors’ accounts in highlighting the role of fear as an accelerator for the separation, which usually occurs soon after the realisation that their life may be in danger.

*SW: Within their heads, but they just suddenly thought, “I can’t live with this guy anymore. He is going to kill me.”* SW6, p. 15, ll. 363–364.

##### Fear for children’s life and safety

Both survivors and support workers assigned a salient role in triggering the separation to the realisation that the abuser may seriously hurt and/or kill the survivor’s children. This accelerator was inherently different from the promoter described above (“desire to protect children from the effects of DA and the Intergenerational Transmission of Violence”). Indeed, mothers’ fear for their children’s life was a far more powerful motivator for leaving, one that often had a direct and identifiable link with the separation, as explained by survivors five and six.

*S*: *He*… *picked up a bottle ermm*… (…) *and he threw it*… *aiming for my son, but he missed him and hit the wall behind us. But*… *because he’d done that, ermm*… *to try and stop him from crying*… *that -obviously in my mind I thought: “I just can’t do that anymore.”* S5, p. 5, ll. 94–102.

*S: I thought*… *it all flashed in my head, I thought: “If he did what he did to me, a grown adult, what the hell could he do to a baby?” And I thought: “Ooooh!’ I felt panic”* (…) *And I thought: “You are not going to do anything to my child”* (…) *And I thought: “That’s it! I’m gonna stop you and save the child as well,” and I did*. S6, pp. 35–36, ll. 802–809.

Support workers also reported mothers’ concerns for their children’s safety as an important factor triggering and/or accelerating the separation process. As support worker three explained:

*SW: It might be* (…*)[that] he’s hurt – he’s hurt one of the children, that might be the trigger*. SW3, p. 33, ll. 885–886.

##### ”This isn’t love”: Changes in the romantic attachment for the perpetrator

An interesting finding from the data was that not all accelerators were directly connected to the abuse. For example, realising that the perpetrator lacked genuine romantic feelings was also a factor related to the survivors’ decision to end the abusive relationship. In the experience of survivor six, understanding that her partner did not feel affection for her and her newborn son represented a pivotal accelerator for the final separation.

*S: And I thought: “He hates us! That’s not love”(*…) *And that was it* (…) *I thought: “This isn’t love.*” S6, p. 35, ll. 796–801.

##### Pressure to leave from services and authorities

This accelerator only emerged in the support workers’ narrations but was the most frequently mentioned. Survivors were often described as leaving their partner due to the pressure of formal sources of support, particularly in situations in which children might be removed from their custody if they remain with the perpetrator. Support worker five described this aspect as follows:

*SW: I think social care getting involved also is a big thing* (…) *If– or other services starting to get involved can* (…) *trigger things. Sometimes that pushes them so that can be the final factor*. SW5, p. 3, ll. 55–61.

Support worker six offered a similar opinion:

SW: *If* (…) *we see the involvement of children’s social care, sometimes that can make a decision for someone. So, for example, if they’re then allocated a social worker or a child protection plan, they might think: “No, I can’t be in this relationship. This needs to end.”* SW6, p. 1, ll. 17–22.

## Discussion

The current study has presented the separation from an abusive partner as resulting from the combined action of two main factors, the “promoters” and the “accelerators.” As discussed above, the “promoters” foster a gradual movement toward the separation stage (increasing victims’ readiness to leave) whilst the “accelerators” act as powerful vectors, accelerating this process. Interestingly, the proposed separation model bears similarities with Newton’s laws of motion (1687, as cited in Haubold and Fairbridge, 1997) and with the first two laws in particular. Indeed, the first law (the “law of inertia”) states that “a body continues in a state of uniform rest or motion *unless acted upon by an external force*” (Haubold and Fairbridge, 1997). There is extensive evidence indicating that DA survivors tend to remain in an abusive relationship due to different barriers to leaving (Dunn, 2005; Eckstein, 2011; Saunders, 2020), and our participants mentioned a wide range of factors motivating survivors’ decision to stay in the abusive relationship. As our findings indicated, a drastic change in staying or leaving decisions emerged as a result of forces that disrupted the *status quo* of the abusive relationship. In this study, we have called these external forces “promoters” and “accelerators” and our findings highlighted their differential influence on the separation process. Newton’s second law states that the acceleration of an object increases if forces are applied and that its acceleration will be directly proportional to the magnitude of the force(s) applied (Haubold and Fairbridge, 1997). As our findings suggested, “promoters” and “accelerators” represent vectors that boost survivors’ leaving decisions and accelerate the separation process. Nevertheless, they emerged as bearing different “magnitudes.” For example, survivors’ realisation that the abuse negatively influenced their children represented a “promoter,” which stimulated reflections on the need to leave the perpetrator. In this sense, this promoter had a “moderate magnitude” (as it increased the likelihood of the separation, but did not directly elicit it). Instead, mothers’ awareness of their children’s life being at risk represented an “accelerator,” bearing a remarkable influence (in our metaphor, “magnitude”) on the separation process. This considered, in our conceptualisation of the separation process, as in Newton’s second law, the process of acceleration (in our case, the journey to leave the perpetrator) is seen as the result of the combined action of different forces (“promoters” and “accelerators”), operating against the resistance to change (Kabe and Sako, 2020). As mentioned above, our participants’ narratives highlighted the presence of multiple factors motivating victims’ decisions to remain in the abusive relationship, and these factors can be seen as the “resistance” to the change brought forward by the joint action of promoters and accelerators. This ongoing dynamic tension between resistance to change and forces promoting it emerged consistently from our participants’ accounts, and clearly outlined the need to abandon models of the separation as a progressive process achieved in multiple sequential stages. Indeed, differently from studies that have adopted the SOC model (Frasier et al., 2001; Cluss et al., 2006; Alexander et al., 2009; Reisenhofer and Taft, 2013), our findings suggest that separation is a non-linear process, with factors and events that might accelerate or decelerate victims’ journey toward the end of the abuse. For example, victims still involved with the abuser (who could, therefore, be in the “precontemplation” stage) might suddenly decide to end the violent relationship. Conversely, victims who carefully planned the separation (going through the “contemplation” and “action” stage) might decide to stay or return to the perpetrator after a temporary separation. Similarly, our findings also suggest the need to go beyond views of the separation process as the result of single “turning points,” i.e., changes occurring in survivors’ lives at a specific time which ultimately lead them to leave the perpetrator (Chang et al., 2006; Enander and Holmberg, 2008; Murray et al., 2015). As we suggested elsewhere (Di Basilio et al., 2021), a perspective of complexity is needed in order to understand and effectively tackle complex forms of trauma (such as living in an abusive relationship). This entails considering the leaving process as the result of forces (“promoters” and “accelerators”) in a state of dynamic tension with centripetal forces promoting the survivors’ permanence in the abusive relationship. Conceiving the separation process adopting a complex perspective may also positively influence current practices to help survivors. For example, professionals using the SOC model might follow guidelines on how to support victims depending on the stage of the separation they find themselves in Frasier et al. (2001). This might lead them to overlook important cognitive, emotional and situational factors operating as “promoters” and “accelerators.” Hence, we advocate the need to go beyond the focus on single “turning points” and to also abandon the aim of shaping “appropriate interventions that best fit with the TTM stage of change” (Catallo et al., 2012, p. 8). Indeed, our study underlined the importance of shaping support interventions based on a complex evaluation of different psychological and situational factors that dynamically interact during the separation phase. This is in line with recently emerging literature indicating the importance of building survivor-centred interventions (Cattaneo and Goodman, 2015; Goodman et al., 2016). Moreover, our findings offer support to the need for DA professionals and policymakers to assign greater importance to the specific factors propelling victims’ decision to leave and the dynamic tension with forces promoting their permanence in the abusive relationship. Lastly, our study indicated a general concordance in the views of survivors and support workers. Nevertheless, survivors mostly described themselves as proactive in achieving and maintaining the separation from their abusive partners. They acknowledged that they benefited from the support of formal and informal sources of help but described the separation process as ultimately led by their deliberate decisions. On the contrary, for support workers, survivors often need to be “guided” through the process of separation, as they are reluctant to leave the perpetrator. According to their narratives, authorities (e.g., police and justice system), services (e.g., social services) and most of all, DA organisations play a key role in allowing survivors to escape the abuse. Hence, victims were usually portrayed by the support workers as passive in achieving the separation, often in need of being prompted about the “right course of action” to permanently end the abuse. Both the survivors’ and the support workers’ conceptions are likely to be the product of meaning-making processes linked to their personal experiences of the separation process (as DA survivors or professionals). Moreover, DA research suggests that the experience of DA victimisation is often associated with feelings of vulnerability and disempowerment (McDermott and Garofalo, 2004; Bell, 2007; Matheson et al., 2015). Therefore, it is possible that the survivors interviewed might have downplayed the importance of formal sources of support in their leaving and staying away decisions. This might have been motivated by the attempt to offer an image of themselves that reflects the empowerment, sense of control and self-confidence matured after the separation. Conversely, support workers’ experiences with DA victims might have contributed to the development of a conception of victims as in need to be supported and guided throughout and after the separation process. Future research must explore further whether the different views held by support workers and survivors influence the help that the latter receive during the separation process. If support workers conceive the role of formal support as essential for survivors, they might focus on promoting victims’ engagement with authorities and DA services, potentially overlooking subjective factors (e.g., survivors’ change in romantic feelings for the partner) that this study outlined as salient in the separation process.

## Limitations

Our participants represent a diverse but not necessarily representative sample of DA survivors and professionals. Moreover, before and during the study, participating survivors received different types of support (e.g., counselling and self-help groups), which may have affected their evaluation of the factors promoting and triggering/accelerating the separation from their abusive partners. Finally, the current study exclusively focused only on female victims of DA. Addressing male victims’ views about the promoters and accelerators for leaving the abusive partner is an important direction for future research.

## Data availability statement

The datasets presented in this article are not readily available because the authors do not have permission to share the dataset (as this was not indicated in the protocol that received ethical approval). Requests to access the datasets should be directed to DDB, d.di-basilio@mmu.ac.uk.

## Ethics statement

The studies involving human participants were reviewed and approved by Manchester Metropolitan University Ethics Committee. The patients/participants provided their written informed consent to participate in this study.

## Author contributions

DDB acted as Principal Investigator for this study, collecting and analysing data and elaborating the conceptual model proposed. ML provided invaluable guidance and support during the research process, whilst FG offered crucial contributions to the conceptual analysis and elaboration of the findings. All authors listed have made a substantial, direct, and intellectual contribution to the work, and approved it for publication.

## Footnotes

1 Although multiple terms (e.g., gender-based violence, intimate partner abuse) are commonly used to refer to domestic violence, the term “DA” will be used throughout the current paper to refer to abuse (in any form) perpetrated by an intimate partner. This term was chosen to be consistent with the United Kingdom cross-government definition of intimate partner abuse as “DA,” as the study this paper refers to was conducted in the United Kingdom.

2 Throughout this paper, both terms “survivors” and “victims” will be used interchangeably. Some evidence suggests that the term “victim” might be associated with conceiving the victimised individual as weak and disempowered (Donovan and Hester, 2010; Murray and Graves, 2013). In contrast, the term “survivor” is often associated with resilience and personal transformation, sometimes even resulting in triumphalist attitudes exalting the concepts of strength and survivorship (Coreil et al., 2012; Dyer, 2015). Despite these semantic differences, people who have experienced DA may define themselves as victims, survivors, both or neither (Hockett et al., 2014; Hockett and Saucier, 2015). Furthermore, these identity aspects can fluctuate depending on personal preferences, contexts and situations (e.g., “victim” in formal circumstances but “survivor” in interpersonal situations; Hockett et al., 2014; Hockett and Saucier, 2015). Therefore, using both terms symbolises the intention to acknowledge both sides (victimisation and survivorship) of DA survivors’ experiences to recognise their complexity and avoid simplistic and unilateral definitions.
